# The management of bipolar mania: a national survey of baseline data from the EMBLEM study in Italy

**DOI:** 10.1186/1471-244X-7-33

**Published:** 2007-07-19

**Authors:** Cesario Bellantuono, Alessandra Barraco, Andrea Rossi, Iris Goetz

**Affiliations:** 1 Department of Medicine and Public Health, Section of Psychiatry and Clinical Psychology, University of Verona, Italy; 2 Eli Lilly Italia S.p.A, Italy; 3 Eli Lilly and Company, Windlesham, UK

## Abstract

**Background:**

Although a number of studies have assessed the management of mania in routine clinical practice, no studies have so far evaluated the short- and long-term management and outcome of patients affected by bipolar mania in different European countries.

The objective of the study is to present, in the context of a large multicenter survey (EMBLEM study), an overview of the baseline data on the acute management of a representative sample of manic bipolar patients treated in the Italian psychiatric hospital and community settings. EMBLEM is a 2-year observational longitudinal study that evaluates across 14 European countries the patterns of the drug prescribed in patients with bipolar mania, their socio-demographic and clinical features and the outcomes of the treatment.

**Methods:**

The study consists of a 12-week acute phase and a ≤ 24-month maintenance phase. Bipolar patients were included into the study as in- or out-patients, if they initiated or changed, according to the decision of their psychiatrist, oral antipsychotics, anticonvulsants and/or lithium for the treatment of an episode of mania.

Data concerning socio-demographic characteristics, psychiatric and medical history, severity of mania, prescribed medications, functional status and quality of life were collected at baseline and during the follow-up period.

**Results:**

In Italy, 563 patients were recruited in 56 sites: 376 were outpatients and 187 inpatients. The mean age was 45.8 years. The mean CGI-BP was 4.4 (± 0.9) for overall score and mania, 1.9 (± 1.2) for depression and 2.6 (± 1.6) for hallucinations/delusions. The YMRS showed that 14.4% had a total score < 12, 25.1% ≥ 12 and < 20, and 60.5% ≥ 20. At entry, 75 patients (13.7%) were treatment-naïve, 186 (34.1%) were receiving a monotherapy (of which haloperidol [24.2%], valproate [16.7%] and lithium [14.5%] were the most frequently prescribed) while 285 (52.2%) a combined therapy (of which 8.0% were represented by haloperidol/lithium combinations). After a switch to an oral medication, 137 patients (24.8%) were prescribed a monotherapy while the rest (415, 75.2%) received a combination of drugs.

**Conclusion:**

Data collected at baseline in the Italian cohort of the EMBLEM study represent a relevant source of information to start addressing the short and long-term therapeutic strategies for improving the clinical as well as the socio-economic outcomes of patients affected by bipolar mania. Although it's not an epidemiological investigation and has some limitations, the results show several interesting findings as a relatively late age of onset of bipolar disorder, a low rate of past suicide attempts, a low lifetime rate of alcohol abuse and drug addiction.

## Background

Although recent surveys have assessed the management of mania in routine clinical practice [1-3], no studies with a large sample of patients, have been conducted to evaluate the management of manic patients in terms of clinical (e.g., symptom profile and severity), functional (e.g., work, social relations, health-related quality of life) and economic (e.g., direct and indirect medical costs) outcomes, throughout different countries. Therefore, there is still an opportunity to learn more about how patients with mania are treated in different clinical practice and the extent to which the outcomes obtained in a naturalistic setting compare with those coming from the "efficacy trials".

Moreover, the availability of new drugs for the management of bipolar disorders has widened the therapeutic options for the clinicians, especially in the use of combined therapies and concomitant medications, which may vary across different countries and across different geographical areas within the same country.

The *European Mania in Bipolar Longitudinal Evaluation of Medication *(EMBLEM) study is a study sponsored by Eli Lilly and company that is being conducted to address the need for further information from different practice, concerning the drug treatment of patients with bipolar mania (euphoric and mixed) and their clinical, functional and economic outcomes in the short and long-term. The main focus of EMBLEM is to describe and assess outcomes among patients treated with antipsychotics, mood stabilizers and drug combinations.

The EMBLEM study offers for the first time the opportunity of collecting and analysing in the Italian psychiatric context, clinical and psychosocial data of a large sample of manic bipolar patients and the drug treatment they received in a naturalistic setting.

This study will evaluate long-term effects, such as compliance, length of therapy, tolerability and quality of life, which will be measured in the 24-month maintenance phase of the study. In addition, it will elucidate the attitude of Italian specialists towards maintenance treatment.

## Methods

EMBLEM is a prospective, observational (non-interventional) study, aimed at evaluating approximately 4000 patients across 500 psychiatric sites in 14 European countries (Belgium, Denmark, Finland, France, Germany, Greece, Ireland, Italy, the Netherlands, Norway, Portugal, Spain, Switzerland and UK).

The primary aim of this study was to assess, from the start of a new episode of acute mania and during a follow-up period of 12 months, the pattern of "antimanic" drugs prescribed in a routine clinical practice and the outcomes of patients who received such prescriptions.

The study included the assessment of clinical, functional and socio-economic outcomes of the treatment during the acute episode and in the maintenance phase. Observations took place within the normal pattern of care on six occasions within the Acute Phase (baseline to 12 weeks) and four further occasions in the Maintenance Phase (up to 24 months). The present article is only focused on baseline data collected in bipolar manic patients in 56 Italian sites.

The participating psychiatrists enrolled patients aged ≥ 18 years, who presented within the standard course of care as in-or out-patients for the treatment of acute mania in the context of bipolar disorder, if they initiated or changed oral medication (antipsychotics, anticonvulsants and/or lithium) for the treatment of mania. Patient consent was obtained according to local regulation. Consent of an appropriate legal authority was collected in the case that a patient was not able to provide the informed consent at the time of the baseline data collection.

Patients with a first episode of mania were eligible for inclusion in the study if, in the opinion of the investigator, the patient had a probable diagnosis of bipolar disorder at the time of the start or change with an oral medication.

As regulatory guidelines are different through European countries participating in the EMBLEM study, where investigators were asked to include in the study half of patients initiated or changed to olanzapine [4], the Italian (and Danish) patients were selected by investigators with no restrictions regarding treatment. Participating investigators were asked to take treatment decisions, including the initial oral treatment change, independently from participation in the EMBLEM study.

Participating patients could receive an add-on therapy, according to standard care of acute mania. Patients who had medications changed or stopped at any time after the baseline observation were kept in the study, as drug discontinuation was not a criterion for study withdrawal.

Baseline data collection was performed within 24 hours from the first change or start with an oral medication during the patient admission or in the outpatient setting. The acute phase of the study included data collection after 1, 2, 3, 6 and 12 weeks after baseline, whereas observations during the follow-up took place at 6, 12, 18 and 24 months.

If an inpatient was discharged before or at 12 weeks, then a discharge observation and data collection was completed within 24 hours. Observations for patients who had been discharged prior to 12 weeks, but who were then readmitted before completion of the 12-week acute phase, continued according to the normal schedule, whenever possible.

The acute phase included the following measures: demographic data, psychiatric history, functional health status, clinical condition, drug already received and prescribed at baseline, concomitant medications, and quality of life. Other outcome measures at baseline included adverse reactions caused by therapies taken prior to enter the EMBLEM study, medical resource use and healthcare costs of the prescribed drugs.

Baseline socio-demographic, psychiatric history and clinical data were collected to characterize the patient sample and detect initial differences between the prescribed therapies. The main outcome variable of the study is the Clinical Global Impression – Bipolar Disorder (CGI-BP), which is a CGI scale modified specifically for use in assessing global illness severity and change in patients with bipolar disorder [5]. The CGI-BP overall, mania, depression and hallucinations/delusions were assessed using a 1–7 scoring rate for severity. Symptoms of mania and depression in the acute phase were measured using the Young Mania Rating Scale (YMRS) [6] and 5-item (i.e. depression, dysphoria, hedonism, psychosis and activation) Hamilton Depression Rating Scale (HAM-D) [7]. The Life Chart Methodology [8] was used in the 4 weeks preceding study entry and throughout the study. Two individual items from the Slice of Life [9] measured the patient outcomes in terms of work functioning and life satisfaction.

Investigators and sites were selected on the basis of their willingness to take part in the study, among those operating in a wide range of treatment services, facilities and locations (e.g. urban and rural, or Northern, Central and Southern Italy). The case record forms permitted a simple and easy data collection in order that observations could be easily integrated into the daily practice of the participating physicians. The collected data were sent by each local investigator to the country coordinator, prior to be forwarded to the Data Entry and Management Centre in Madrid, Spain.

In addition to the recruitment of investigators, additional clinical, research and policy experts took part in the study via a European Advisory Board, which included at least one non-sponsor representative of each participating country.

## Results

### Demographics

Five hundred and sixty-three patients (305 females, 254 males and 4 with missing data) were recruited in a total of 56 Italian sites. Of these, 376 (66.8%) were outpatients and 187 (33.2%) were inpatient. The mean age was 45.8 (± 13.5) years in the total population (range 18–79), 46.9 (± 13.5) years in females and 44.5 (± 13.4) years in males; the corresponding values of BMI were 26.7 (± 5.4), 26.5 (± 5.5) and 27.0 (± 5.2 kg/m^2^), respectively. Five patients did not have any education, 119 (21.3%) completed the primary school, 246 (44.1%) the lower/upper secondary school, and the remaining had a post-secondary vocational training or a University degree (information is missing in 5 patients). The vast majority of patients were outpatients (67%); no formal comparisons between hospitalized and outpatients were performed.

### Psychiatric history

Psychiatric history and admission details of the sample are briefly summarised in Table 1. The mean age of onset of symptoms was 29.7 (± 11.1) years and the mean age at first treatment was 31.3 (± 10.9) years.

**Table 1:** Patients' psychiatric history

**Characteristic**	**Results**
Age at onset of mood symptoms, *mean *± *SD (range)*:	
Age at first symptoms of bipolar disorder	29.7 ± 11.1 (2–77) years
Age at onset of manic or mixed episodes	31.4 ± 11.8 (2–77) years
Age at onset of depressive episodes	31.3 ± 11.9 (0–74) years
Age at start of treatment *mean *± *SD (range)*:	
Age at first treatment of mood symptoms	31.3 ± 10.9 (13–74) years
Age at first contact with psychiatric services	32.9 ± 12.0 (13–74) years
Age of first admission for psychiatric symptoms	32.9 ± 12.2 (0–79) years
Psychiatric services used in the past 12 months, *mean *± *SD (range)*:	
Number of admissions due to bipolar disorders	1.1 ± 3.8 (0–61)
Number of days in inpatient facility	10.7 ± 20.3 (0–148)
Number of days in day care	5.2 ± 25.0 (0–300)
Number of outpatients consultations	10.6 ± 13.5 (0–100)
Number (%) of Alcohol users/abusers/dependents.	
Users	129 (23.3)
Abusers	42 (7.6)
Dependant	4 (0.7)
Number (%) of Cannabis: users/abusers/dependents	
Users	30 (5.5)
Abusers	13 (2.4)
Dependant	2 (0.4)
Number (%) of other substances users/abusers/dependents	
Users	8 (1.4)
Abusers	10 (1.8)
Dependant	2 (0.4)

The present was the first episode in 32 patients (6.6%). The number of patients that exhibited 1, 2, 3 and > 4 manic or mixed episodes in the past 12 months was 255 (45.6%), 175 (31.3%), 59 (10.6%) and 42 (7.5%), respectively; the information was unknown in 32 patients. A total amount of 254 patients (45.4%) did not have depressive episodes in the past 12 months, while 173 (30.9%) had one episode and 85 (15.2%) more than one. One manic-mixed depressive episode was experienced by 138 (27.6%) patients, while 362 (72.4%) had more than one episode in the past 12 months (104 had > 4 episodes). The number of patients with rapid cycles in the previous 12 months was 104 (20.8%).

A total of 117 patients (21.1%) attempted suicide in the past, 26 (4.7%) of them in the previous 12 months (9 more than once). Details on alcohol/cannabis/drug abuse are shown in Table 1. In the past, 105 (19.0%) patients had alcohol problems, 57 (10.2%) had problems with cannabis and 27 (4.8%) with other substances.

### Social status

A number of 231 patients (41.1%) were single, while 260 (46.3%) were married (whereby 5.7% were not living together) and 71 (12.6%) had a partner. Most of patients lived in independent residence (n = 245, 43.6%) or in a residence as dependent family member (n = 279, 49.6%). A total of 121 patients (21.5%) never took part in social activities in the previous 4 weeks, 58 (10.3%) took part once and the rest (68.1%) more than once. Eighty-seven patients (15.5%) had no impairment in work activities, while the level of impairment was mild in 84 (15.0%), moderate in 201 (35.8%) and severe in 71 (12.7%); 100 (17.8%) were unable to work due to mental illness. Thirty-three patients (5.9%) were very satisfied with his/her life, 126 (22.5%) were satisfied, 171 (30.5%) were neither satisfied nor dissatisfied, 169 (30.2%) were dissatisfied and 61 (10.9%) were very dissatisfied.

### Clinical status

The mean overall CGI-BP score in the past year was 4.2 (± 1.1). The mean score at baseline was 4.4 (± 0.9) both in overall and mania, 1.9 (± 1.2) in depression, and 2.6 (± 1.6) in hallucinations/delusions (which were reported in 202 patients, 36.8%). The number and proportion of patients who were markedly, severely or very severely ill at baseline was 235 (42.0%) in total score, 244 (43.6%) in the mania score, 15 (2.7%) in the depression score and 86 (15.3%) in the hallucinations/delusions score. The duration of the episode of mania at baseline was < 1 week in 157 patients (28.1%), 1–2 weeks in 188 (33.6%), 3–4 weeks in 99 (17.7%), 5–8 weeks in 61 (10.9%), and > 8 weeks in 54 (9.7%). The results of the YMRS single items are presented in Table 2. A total of 81 patients (14.4%) had a total score < 12, 141 (25.1%) had a score ≥ 12 and < 20, 340 (60.5%) had a score ≥ 20 (one patient was not evaluated). The mean total score of HAM-D was 3.0 (± 2.5). The results of the Life Chart Method in the week prior to enter the study showed that the mean values were 5.7 (± 2.7) for mania, 0.5 (± 1.4) for depression and 5.9 (± 2.5) as overall score.

**Table 2:** Score of Young Mania Rating Scale by single items

**Item**	**Mean ± SD (range)**
Elevated mood	2.2 ± 1.1 (0–4)
Increased motor activity-energy	2.3 ± 1.0 (0–4)
Sexual interest	1.1 ± 1.1 (0–4)
Sleep	2.1 ± 1.2 (0–4)
Irritability	3.2 ± 1.9 (0–8)
Speech (rate and amount)	3.3 ± 1.9 (0–8)
Language-thought disorder	1.5 ± 0.9 (0–4)
Content	3.1 ± 2.5 (0–8)
Disruptive-aggressive behaviour	1.5 ± 1.6 (0–8)
Appearance	1.2 ± 1.1 (0–4)
Insight	1.6 ± 1.4 (0–4)

Total score	22.9 ± 10.2 (4–57)

### Medication's prescription at baseline

In the past 4 weeks prior to baseline, 316 patients (56.2%) were fully compliant to prescribed medication, 140 (24.9%) were compliant approximately half of time, 55 (9.8%) were almost never compliant and 51 (9.1%) were not on drugs. Lack of efficacy was the main reason for starting with a new medication at baseline and was reported in 317 patients (57.3%). Other reasons were no intake of previous medication (66, 11.9%), poor compliance (50, 9.0%), patient's request (8.0% of patients) and side-effects (5.8%). The most common adverse events reported with treatment taken prior to baseline visit were loss of memory/difficulties in concentration (in 245 patients, 43.6%), akatisia (in 231 patients, 41.3%), sedation (in 207, 36.8%) and insomnia (in 201, 35.8%).

**Figure 1:**
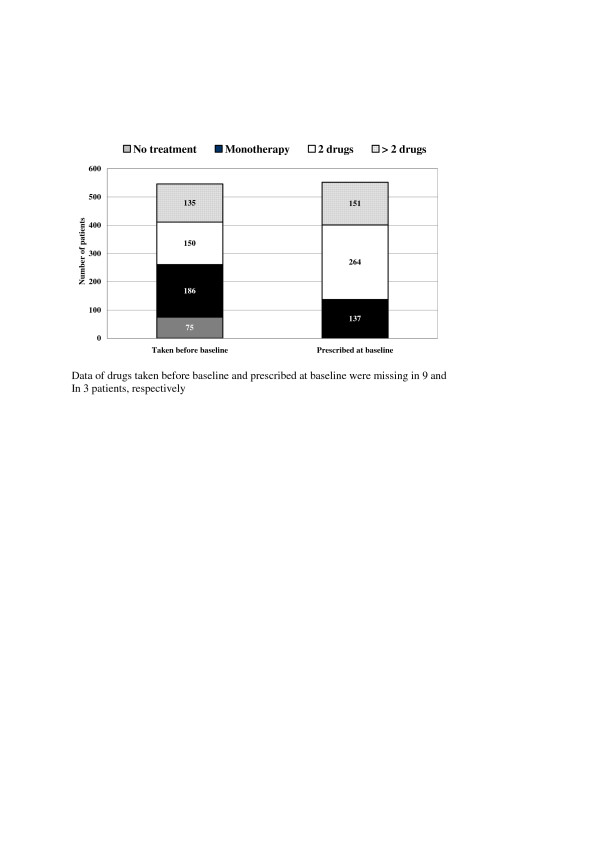
Distribution of patients according to the number of psychotropic drugs taken before/prescribed at baseline. Data of drugs taken before baseline and prescribed at baseline were missing in 9 and in 3 patients, respectively.

The distribution of patients according to the number of psychotropic drugs taken before baseline and prescribed at baseline is shown in Figure 1. The most common drugs taken before baseline in each class (monotherapy, co-therapy, more than two drugs) are presented in Table 3. Seventy-five patients (13.7%) were naïve to treatment. Among patients receiving monotherapy before baseline (n = 186, 34.1%), 51 (27.4%) were treated with atypical antipsychotic agents, 63 (33.9%) with typical agents, 45 (24.1%) with other anticonvulsants, and 27 (14.5%) with lithium. The most common combinations (n = 285, 52.2%) were typical + anticonvulsants (in 55 patients, 19.3%), atypical + anticonvulsants (in 40, 14.0%), typical + atypical + anticonvulsants (in 30, 10.5%), typical + lithium (in 28, 9.8%) and typical + lithium + anticonvulsants (in 27, 9.5%). With regards to intramuscular therapies, 96 patients (12.3%) were taking fast-acting typical agents, 19 (3.4%) fast-acting atypical agents, 61 (11.0%) benzodiazepines and 9 (1.6%) other fast-acting medications.

**Table 3:** Drug treatment registered at baseline (before change) by class

**Monotherapy (n = 186)**		**Co-therapy with 2 drugs (n = 150)**		**Polytherapy (> 2 drugs) (n = 135)**	
Drug	N (%)	Drugs	N (%)	Drugs	N (%)

Haloperidol	45 (24.2)	Other typical/atypical	15 (10.0)	Haloperidol/other typicals/other AC	11 (8.2)
Valproate	31 (16.7)	Haloperidol/Lithium	12 (8.0)	Haloperidol/other typicals/Valproate	11 (8.2)
Lithium	27 (14.5)	Olanzapine/Valproate	10 (6.7)	Haloperidol/other typicals/Lithium	8 (5.9)
Olanzapine	19 (10.2)	Risperidone/non-valproate AC	10 (6.7)	Other typicals/other AC/Lithium	8 (5.9)
Other typical	18 (9.7)	Other typical/Valproate	10 (6.7)	Haloperidol/other typicals	3 (2.2)
Risperidone	16 (8.6)	Risperidone/Lithium	9 (6.0)	Other typicals/Valproate/Lithium	2 (1.5)
Other AC	14 (7.6)	2 typicals	9 (6.0)	Other combinations	92 (68.1)
Quetiapine	10 (5.4)	Other typical/Lithium	8 (5.3)		
Other atypical	6 (3.2)	Haloperidol/Valproate	8 (5.3)		
		Lithium/non-valproate AC	8 (5.3)		
		Olanzapine/Lithium	7 (4.7)		
		Haloperidol/non-valproate AC	7 (4.7)		
		Other combinations	37 (24.7)		

AC = Anticonvulsivants;

The most common drugs prescribed at baseline in each class are presented in Table 4. Among prescribed monotherapies (n = 137, 24.8%), 87 patients (63.5%) received atypical antipsychotic agents, 10 (7.3%) typical agents, 31 (22.6%) other anticonvulsants, and 9 (6.6%) lithium. The most commonly prescribed combinations (n = 415, 75.2%) were atypical + anticonvulsants (in 147 patients, 35.5%), atypical + lithium (62, 14.9%), typical + atypical + anticonvulsants (in 43, 10.4%) and atypical + lithium + anticonvulsants (in 34, 8.2%).

**Table 4:** Drug treatment prescribed at baseline (after change) by class

**Monotherapy (n = 137)**		**Co-therapy with 2 drugs (n = 264)**		**Polytherapy (> 2 drugs) (n = 151)**	
Drug	N (%)	Drugs	N (%)	Drugs	N (%)

Olanzapine	69 (50.4)	Olanzapine/Valproate	59 (22.3)	Olanzapine/Valproate/Lithium	11 (7.3)
Valproate	21 (15.3)	Olanzapine/Lithium	39 (14.8)	Olanzapine/other AC/Lithium	10 (6.6)
Risperidone	11 (8.0)	Olanzapine/non-valproate AC	34 (12.9)	Olanzapine/other typicals/Valproate	8 (5.3)
Other AC	10 (7.3)	Risperidone/Valproate	19 (7.2)	Olanzapine/other typicals/other AC	7 (4.6)
Lithium	9 (6.6)	Other typical/atypical	16 (6.1)	Olanzapine/other typicals/lithium	2 (1.3)
Quetiapine	6 (4.4)	Risperidone/Lithium	14 (5.3)	Other combinations	113 (74.8)
Haloperidol	6 (4.4)	Other atypical/Valproate	11 (4.2)		
Other typical	4 (2.9)	Haloperidol/Lithium	9 (3.4)		
Other atypical	1 (0.7)	Other atypical/non-valproate AC	7 (2.7)		
		Other typical/Lithium	7 (2.7)		
		2 typicals	7 (2.7)		
		Other typical/non-valproate AC	6 (2.3)		
		Risperidone/non-valproate AC	6 (2.3)		
		Other combinations	30 (11.4)		

AC = Anticonvulsivants;

Antidepressants were taken prior to baseline by 135 patients (24.7%) as SSRI, by 36 (6.58%) as TCAs and by 16 (2.9%) in form of other medications; the corresponding prescribed numbers were 71 (13.0%), 7 (1.3%) and (0.7%). Benzodiazepines were taken prior to baseline by 379 patients (69.3%) and were then prescribed in 326 (59.2%).

## Discussion

The EMBLEM study is the first prospective observational survey focusing on the patterns of drugs prescribed, the therapeutic management and the outcomes of a large sample of patients affected by bipolar mania in 14 European countries [4]. In particular, the Italian cohort may be particularly interesting as it represents a "more naturalistic" sample of patients treated in routine clinical practice, because no restricted treatment assignment was adopted in the protocol.

The study was designed with no restrictive inclusion and exclusion criteria in order to have an as much as possible wide representation of patients receiving treatment for acute mania in the context of bipolar disorder. The EMBLEM provides relevant clinical and epidemiological information on the psychotropic drugs treatment prescribed in a real-world situation to bipolar in- and outpatients as well as on the effectiveness of these treatments at baseline and during a follow-up of 24 months.

A previous survey performed in Europe that involved more than 10 countries, the GAMIAN-Europe/BEAM Survey [10], showed some similarities in problems and difficulties encountered in the management of bipolar patients across different countries, regardless of the political, social or cultural settings. Social functioning and integration of patients with bipolar disorders were shown to be still poor and education of bipolar patients about drug treatment and psychosocial interventions were found to be still a healthcare priority [11]. However, the relatively low amount of patients observed, which provided little country-specific information, and the lack of longitudinal data over time represented relevant limitations of the study.

The baseline data coming from the Italian cohort of the EMBLEM study have shown some relevant information about the characteristics and the pattern of drug used in patients with acute bipolar mania. Most of the subjects observed (66.8%) were outpatients and about half had a good educational level. Data of onset of psychiatric symptoms showed that manic or mixed and depressive episodes started at a similar mean age. A recent survey of the Stanley Foundation Bipolar Network (SFBN) that included bipolar patients [12] showed an earlier age at onset compared to that of the present study (i.e. approximately 21 vs. 30 years on average) and that age of onset of depression preceded the date of onset of mania or mixed states of 4 years on average. In the view that the mean age at first onset generally reflects the polarity of the disorder, as shown in a recent retrospective analysis [13], the similar mean age at onset of manic/mixed and depressive symptoms found in this study may be as indicator of a lack of predominance of one pole over the other in our sample.

Only a minority of patients attended the clinics at the first episode, while approximately half of them had at least 2 manic or mixed episodes and more than 70% had manic, mixed or depressive episodes in the last 12 months. The time from the first onset of symptoms of bipolar disorder to the start of treatment was relatively short (on average 18 months); however, the first contact with a psychiatric service was taken on average 18 months after a treatment was started, presumably in a non-psychiatric setting. The lag time from the first symptoms and treatment was markedly lower than that found in the survey of the SFBN, in which first treatment for bipolar patients had been delayed by an average of 10 years from illness onset [14].

The analysis of co-morbidities showed that substance-use disorders, which are frequently associated with mood disorders and may negatively influence their course and outcome [15,16], were reported in a relatively small amount of subjects. Consistently with other reports [17], approximately one fifth of patients had alcohol-correlated disorders in the past and less than 10% were abusers or dependent at study entry. This relatively low amount of substance-abuse disorders is likely to be the result of the healthcare organization in Italy, which includes specific services dedicated to drugs users and hence a lower referral to psychiatric services.

A total number of 117 patients (21.1%) attempted suicide, and 26 (4.7%) in the previous 12 months. The risk of suicide appears to be in line with findings of some surveys [18] but much lower than that reported in others [19], possibly due to the relatively late onset of bipolar disorders in this population. Consistently with the results reported by the SFBN [14], patients admitted to this study had severe limitation of social and occupational functioning: 48.3% had moderate or severe impairment of occupational activities and a further 17.8% was totally unable to work due to mental illness.

The distribution of CGI-BP also showed a high level of impairment. The mean baseline mean score of mania (4.4) was higher than the score of depression (1.9) and of hallucinations/delusions (2.6). Accordingly, 60.5% of patients had an YMRS score ≥ 20. The main reason of the start/change of prescription were lack of efficacy of the ongoing therapy (57.3% of patients), which accounted for lack of compliance and the need for alternative treatment in a significant proportion of patients. A high rate of patients also reported poor tolerability of the current therapies, which were taken in 86.3% of patients.

The start/change of prescription at baseline led to a low number of patients with prescribed monotherapy (24.8% vs. 34.1%) and to an increasing of combined therapies, with 75.2% of patients with prescribed combinations compared to 52.2% of patients taking combined therapies at baseline. Among patients with monotherapy, prescription of atypical antipsychotics increased from 27.4% of patients to 63.5%, while typical agents decreased from 33.9% to 7.3%; prescription of lithium and other anticonvulsants as single agent was also reduced.

The most commonly antipsychotics prescribed in combination, were represented by atypical agents. The prescription of olanzapine in monotherapy increased from approximately 10% to 50% of patients, and also was the most prescribed agent both in co-therapy (in 22.3% of patients with valproic acid, in 14.8% with lithium and in 12.9% with other antimanic drugs) and in polytherapy with 3 or more drugs. The prescription of antidepressants and to a lesser extent of benzodiazepines, decreased compared to pre-baseline condition.

Therefore, based on the patients' baseline condition, substantial changes of drug treatment were reported. A wide spectrum of therapies was prescribed in patients assigned to dual therapy and, in a greater extent, in those prescribed with 3 or more drugs.

This survey presents some limitations: this study included patients who needed to initiate or to change their drug treatment due to the acute episode of mania; respect to this, the reliability of CGI-BP to assess the symptoms in the past year may be questionable. Furthermore, our sample size could be not adequately powered to draw reliable conclusion on effects of drugs prescribed in the longitudinal phase of the study in the view of the high variability of treatments.

## Conclusion

The baseline data collected in the Italian cohort of patients taking part in the EMBLEM study showed a high degree of clinical and functional impairment at baseline and great and variable changes in drug prescriptions. However, patients enrolled in Italy represent an adequate sample to address country-specific information on clinical, social and economic outcomes in the management of acute bipolar mania. The analyses of the follow-up data concerning the management of the bipolar patients from the Italian cohort of the EMBLEM study are still in progress.

## Competing interests

This study was sponsored by Eli Lilly and company. Andrea Rossi, Alessandra Barraco and Iris Goetz are currently working for Eli Lilly.

Cesario Bellantuono has received honoraria for educational CME activities in the field of Clinical Psychopharmacology from Eli Lilly, AstraZeneca, Bristol Myers Squibb, Lundbeck, GSK, Pfizer, Jansenn-Cilag, Boeringher-Ingheleim.

According to Italian law, honoraria for recruiting patients were paid to participating Institutions.

## Authors' contributions

Andrea Rossi, Alessandra Barraco and Iris Goetz have given substantial contributions to conception and design, to acquisition of data, and to analysis and interpretation of data. They have been involved in revising the manuscript and have given final approval of the version to be published. Cesario Bellantuono has given relevant contribution in the recruitment of patients and in the critical revision of the manuscript and has given final approval of the version to be published.

## Pre-publication history

The pre-publication history for this paper can be accessed here:


